# Office-Based Structural Autologous Fat Injection Laryngoplasty for Unilateral Vocal Fold Paralysis

**DOI:** 10.3390/jcm11164806

**Published:** 2022-08-17

**Authors:** Andy Wei-Ge Chen, Chih-Hua Chen, Tsai-Ming Lin, Angela Chih-Hui Chang, Tzu-Pei Tsai, Shyue-Yih Chang

**Affiliations:** 1 Department of Otorhinolaryngology, Head and Neck Surgery, Changhua Christian Hospital, Changhua 500, Taiwan; 2 Charming Institute of Aesthetic and Regenerative Surgery, Kaohsiung 807, Taiwan; 3 Department of Plastic Surgery, Kaohsiung Medical University, Kaohsiung 807, Taiwan; 4 Voice Center, Department of Otolaryngology, Cheng Hsin General Hospital, Taipei 114, Taiwan; 5 Department of Speech, Language and Hearing Sciences, Indiana University Bloomington, Bloomington, IN 47408, USA

**Keywords:** office-based, injection laryngoplasty, unilateral vocal paralysis, autologous fat

## Abstract

Unilateral vocal fold paralysis (UVFP) is a common cause of incomplete glottic closure, leading to significant somatic and social disabilities. Office-based autologous fat injection laryngoplasty (AFIL) has been proposed as an effective treatment for glottic insufficiency but has not been well-studied for UVFP. We enrolled 23 patients who underwent office-based structural AFIL due to unilateral vocal paralysis at our institution between February 2021 and January 2022. In the procedure, autologous fat was harvested and injected into the vocal fold under the guidance of flexible digital endoscopy for structural fat grafting. The voice handicap index-10 (VHI-10) score and perceptual voice measurements were collected before the operation, 2 weeks postoperatively, and 3 months postoperatively. Twenty-two patients were followed-up for at least 3 months. The VHI-10 score improved significantly from 29.65 ± 8.52 preoperatively to 11.74 ± 7.42 at 2 weeks (*p* < 0.0001) and 5.36 ± 6.67 at 3 months (*p* < 0.0001). Significant improvements in grades of dysphonia (*p* < 0.0001), breathiness (*p* < 0.0001), and asthenia (*p* = 0.004) were also noted at 3 months postoperatively when perceptual measurements were investigated. Office-based structural AFIL is an effective treatment for improving voice-related disability for UVFP patients.

## 1. Introduction

Glottic closure is a critical component of phonation and swallowing [1]. Unilateral vocal fold paralysis (UVFP) is a common cause of incomplete glottic closure. UVFP is commonly related to surgical complications, malignancy invading the recurrent laryngeal nerve, or other idiopathic etiologies [2,3]. The impact of UVFP extends beyond somatic symptoms, causing patients to experience frustration, isolation, fear, and alternations in self-identity [4]. This will significantly affect patients’ quality of life [5,6], leading to disabilities in social relationships and professional careers [7].

Several surgical techniques have been developed to treat UVFP, including injection laryngoplasty, laryngeal framework surgery, and recurrent laryngeal nerve reinnervation [8]. Laryngeal framework surgery, also known as thyroplasty type I, is usually arranged under local anesthesia in the operating room. Various materials, such as silicone, Gore-Tex [9], or autologous thyroid cartilage [10], can be implanted into the larynx through a small skin incision to provide vocal fold medialization. Injection laryngoplasty, on the other hand, can be arranged in office-based situations. Various materials, such as autologous fat, hyaluronic acid [11,12], or calcium hydroxylapatite [13], can be delivered into the thyroarytenoid muscle through injection needles. Similar results [14,15] have been observed for laryngeal framework surgery and injection laryngoplasty. Laryngeal framework surgery provides the opportunity for arytenoid adduction for selected patients, and injection laryngoplasty allows for vocal fold augmentation as well as medialization.

Introduced in 1999, the distal-chip camera for aerodigestive endoscopy [16] has become a critical tool in laryngeal surgery. Modern commercialized flexible digital nasopharyngoscopes can provide high-definition surgical views with distal-chip cameras. Without sedation, laryngeal gargling with 4% topical lidocaine can offer a stable operation field, which is needed for office-based laryngeal procedures. Flexible endoscopic biopsy, vocal fold injection [17], and laser surgeries [18] are well-established procedures performed in office-based settings.

Autologous fat injection laryngoplasty (AFIL) is a well-established procedure for treating vocal fold paralysis [19]. While autologous fat was first known as a transient filler, favorable long-term results have also been reported in the literature [20,21,22]. Coleman [23,24] pointed out that distributing tiny fat parcels in a multilayered fashion may improve long-term fat survival. Although the complication rate of AFIL is low, Sanderson et al. [25] reported a 3.4% rate of poor voice outcome caused by aggressive fat overinjection. A more sophisticated injection method might be helpful in preventing such complications.

While AFIL is traditionally performed in the operating room under general anesthesia, our research team has previously established the application of office-based AFIL for vocal process granuloma [26] and glottic insufficiency [27]. Compared with general anesthesia techniques, office-based AFIL can allow for direct feedback on the patient’s voice performance to be obtained by phonation during the operation. Vocal fold tension under flexible endoscopy is much more natural than that obtained under direct laryngoscope suspension, which is required in general anesthesia techniques. Thus, this approach provides the opportunity for more precise tailoring of the volume and distribution of grafted fat tissue.

To our knowledge, the use of office-based AFIL among those with UVFP has not yet been investigated. The current study aimed to evaluate the improvement in voice disability and perceptual voice quality among UVFP patients after officed-based structural AFIL.

## 2. Materials and Methods

### 2.1. Study Design

This was a single-institution, retrospective study arranged in a tertiary referral medical center. All surgeries were performed by a single surgeon (A.W.G. Chen). Speech therapy and evaluation were arranged by a single speech-language pathologist (C.H. Chen). The study was approved by the hospital’s Institutional Review Board (CCH IRB No. 220407).

### 2.2. Patients

Patients who underwent office-based autologous fat injection laryngoplasty due to unilateral vocal palsy from February 2021 to January 2022 were enrolled in the study. The exclusion criteria were (1) bilateral vocal palsy, (2) laryngeal cancer or hypopharyngeal cancer with a previous surgical history, and (3) previous radiotherapy over the laryngeal area.

The option of office-based AFIL was provided as soon as possible for patients with end-stage cancer and irreversible vocal fold palsy. For other patients, the options of 12 months of watchful waiting, short-lasting injection laryngoplasty, and early AFIL were provided. The probability of recovery and prognosis were thoroughly discussed according to etiology and onset time.

### 2.3. Operation Methods

The procedure was performed in our voice clinic under local anesthesia. Ten milliliters of 1% lidocaine was injected into the lower abdomeninal area. Approximately 5 mL of lipoaspirate was harvested through a 0.3 cm skin incision by a blunt tip 3 mm liposuction cannula connected to a 10 mL Luer-Lok syringe. Low pressure was employed manually during liposuction to minimize the damage to fat tissue. According to Lin et al. [28], pulling back from 2 to 3 mL using a 10-mL syringe and liposuction cannula can provide an ideal negative pressure of from approximately 270 to 330 mmHg.

The lipoaspirate was kept in a 10 mL syringe, which was closed with a centrifugation cap, and then processed by standard centrifugation at 500× *g* for 5 min. The bloody solution in the bottom and oil at the top were removed, leaving only purified fat tissue from the middle portion for transplantation. Subsequently, the purified fat was transferred into a 1 mL syringe. A double-bend 19-gauge needle, as reported in our previous article [27], was then connected to the 1 mL syringe.

**Figure 1:**
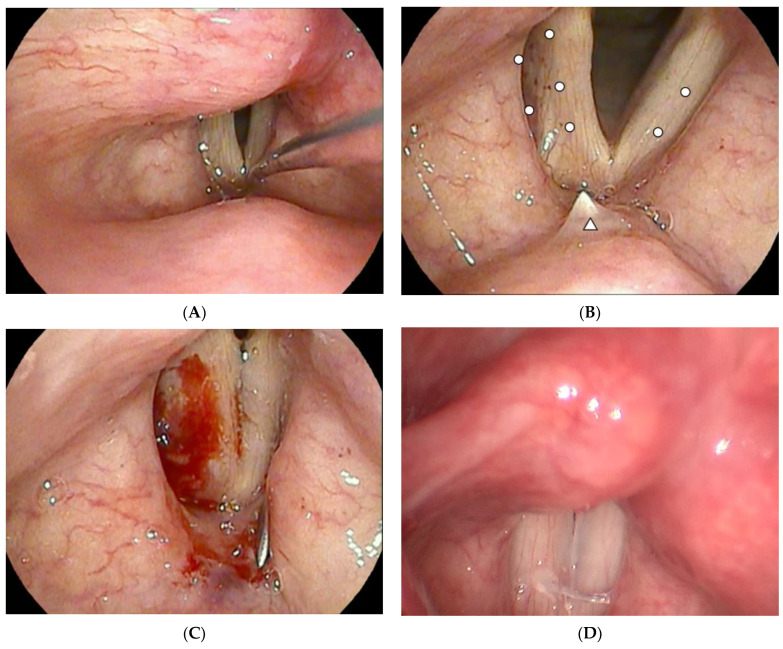
Demonstration of surgery. (**A**) After lidocaine was applied for gargling through epidural catheter, the quality of topical anesthesia could be confirmed by pricking the laryngeal mucosa with the tip of the epidural catheter. (**B**) White dots represent the planned injection points. Tenting of the mucosa can lead the needle to puncturing into the larynx (white triangle). (**C**) Structural autologous fat injection laryngoplasty was performed, and closure of the glottic gap could be identified under digital endoscopy. Bleeding was self-limited. (**D**) Rigid stroboscopy examination at 3 months postoperatively showing closure of the glottal gap.

Local anesthesia was administered with vaporization of 4% lidocaine into the nose and pharynx. The patient was placed in a semi-seated position with the head tilted slightly backward. A flexible digital endoscope (VNL15-J10 and EPK-3000, Pentax Medical) was inserted trans-nasally. For laryngeal gargling, approximately 5 mL of 4% lidocaine was instilled into the larynx through the working channel of the fiberscope using an epidural catheter. The quality of anesthesia by laryngeal gargling of lidocaine was checked by pricking the vocal fold with the tip of the epidural catheter (Figure 1A).

A double-bend 19-gauge needle [27] was delivered into the larynx using the transthyrohyoid approach technique [17]. Purified fat tissue was injected into the vocal muscle, oblong pit of arytenoid cartilage, and paraglottic space to achieve augmentation and medialization (Figure 1B). A small amount of fat tissue could also be injected into the contralateral healthy vocal fold if needed (Figure 1C). Slight overcorrection was performed according to direct phonation feedback during operation. The injection was tailored individually and performed carefully using a multilayered, multipoint method, which is known as the structural fat grafting technique [29]. An example of an office-based structural AFIL procedure is demonstrated in Video S1.

Double-check endoscopy was performed 20 min after the operation. Patients could leave the clinic after that time. To avoid choking due to laryngeal anesthesia, patients were advised to avoid drinking or eating for 2 h. Two days of voice restriction were typically required following surgery.

During the COVID-19 pandemic, patients received polymerase chain reaction tests or rapid antigen tests before surgery. Surgeons used powered air-purifying respirators or N95 respirators and face shields as personal protective equipment during the operation.

### 2.4. Laryngeal and Voice Assessment

Patients were requested to return to our voice clinic at 2 weeks and 3 months postoperatively. Flexible nasopharyngoscopy or rigid video stroboscopy was performed to evaluate vocal fold closure and vibration.

The Voice Handicap Index-10 (VHI-10) questionnaire [30] was administered for patients self-assessment. The VHI-10 score, ranging from 0 to 40, represents voice-related disability, with higher scores indicating higher disability. According to a previous study [31], a VHI-10 score larger than 10 should be considered abnormal. For perceptual voice evaluation, the GRBAS scale [32] was applied by the same speech-language pathologist. The GRBAS scale is a subjective scoring system that includes the grades of dysphonia, roughness, breathiness, asthenia, and strain. A four-point scale is used for each component, with 0 indicating no abnormality, 1 indicating slight abnormality, 2 indicating moderate abnormality, and 3 indicating severe abnormality.

The primary outcome of interest was the VHI-10 score. The secondary outcome was the GRBAS grade. Both evaluations were obtained preoperatively, 2 weeks postoperatively, and 3 months postoperatively.

### 2.5. Statistical Analyses

Data are expressed as the means and standard deviations. The differences in end points were compared with paired samples *t*-test. The Wilcoxon test was performed if the data were not normally distributed. Differences were considered significant when *p* < 0.05. Inter-rater reliability was analyzed by Cohen’s kappa agreement test. Statistical analyses were performed using a commercially available statistical software, MedCalc, version 15.8 (MedCalc Software, Ostend, Belgium).

## 3. Results

Twenty-three patients (mean age 58.65 years; range 30 to 82 years) were enrolled. The characteristics of these patients are shown in Table 1. Thirteen patients suffered from left vocal paralysis, and ten suffered from right vocal paralysis. The most common etiology of vocal paralysis in our cohort was related to thyroid disease and surgery, accounting for 47.8%, and followed by pulmonary and mediastinal disease, accounting for 26.1%.

The number of injection points and volume of injected purified fat tissue on each vocal fold were recorded and are shown in Table 2. To enhance glottic closure [29], 91.3% of patients received contralateral injections. An average of 0.79 ± 0.17 mL of fat tissue was injected into the ipsilateral vocal fold, and 0.19 ± 0.11 mL was injected into the contralateral side. These values might be overestimated since a minimal amount of fat tissue can be discharged after removal of the needle.

An average of 5.35 ± 1.03 points were injected in the ipsilateral vocal fold, with 3.22 ± 0.80 medial points injected and 2.13 ± 0.46 lateral points injected. An average of 2.00 ± 0.74 medial points were injected on the contralateral healthy vocal fold, and no lateral points were injectioned.

All 23 patients underwent the procedure in our voice clinic without inpatient admission. All patients tolerated the procedure well, and no respiratory or neurological complications were noted after the surgery. No swelling or uncontrollable bleeding was noted in the neck or laryngeal area. No postoperative hemorrhage was noted at the donor site, despite the minimal transient bruising detected at the 2-week postoperative follow-up.

**Figure 2:**
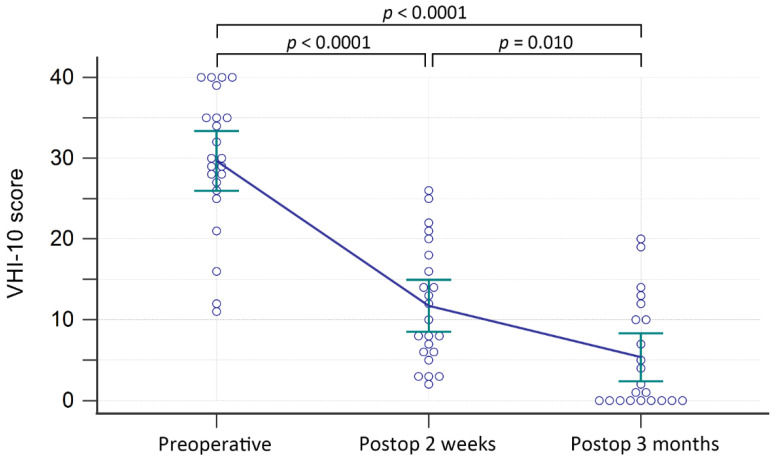
Results of VHI-10 evaluation. Error bars represent the 95% confidence interval of the means. Abbreviation: PostOP, Postoperative.

All 23 patients received speech therapy [33] during follow-up with a median value of 3 sessions (range from 1 to 13; interquartile range, 2.75). Voice quality improved soon after the procedure in all patients. Twenty-two patients were followed up for 3 months. One patient died due to lung cancer before completing follow-up. The results of the VHI-10 scoring evaluation are shown in Figure 2. The VHI-10 score improved significantly from 29.65 ± 8.52 before the operation to 11.74 ± 7.42 at the 2-week follow-up (*p* < 0.0001, 95% CI −20.90 to −14.93). It also improved significantly from 2-week follow-up to 3-month follow-up (11.73 ± 7.59 vs. 5.36 ± 6.67, *p* = 0.010, 95% CI −11.01 to −1.72). Overall, the VHI-10 score improved significantly from preoperatively to 3 months postoperatively (*p* < 0.0001, 95% CI −28.85 to −19.24).

The perceptual parameters of the GRBAS scale are shown in Table 3. A significant improvement from preoperatively to 3 months postoperatively was noted in the grade of dysphonia (*p* < 0.0001, 95% CI −1.51 to −0.77), breathiness (*p* < 0.0001, 95% CI −1.65 to −0.72), and asthenia (*p* = 0.004, 95% CI −0.97 to −0.21). At the end of the study, all voice records were graded again by a second blinded speech-language pathologist, revealing a strong inter-rater reliability (kappa = 0.89).

## 4. Discussion

The concept of structural fat grafting was proposed by Coleman [24] in 1994 and has since been widely accepted by plastic surgeons. He emphasized that minuscule parcels of fat tissue should be grafted in a multilayered manner. Each parcel of fat tissue should be at least as small as 0.1 mL and should ideally be smaller than 1/30 mL in areas of thinner skin such as the eyelid. According to Coleman, separation of the tiny parcels of fat maximizes contact between the surfaces of the transplanted fat and surrounding recipient tissues to encourage integration, anchorage, and long-term survival [23]. More recently, Eto et al. [34] demonstrated three zones from the periphery to the center of grafted fat tissue in a mouse model. In the surviving zone, only adipocytes located within 300 μm of the fat parcel edge survived. In the second zone, the regenerative area, adipose-derived stromal cells survived, and dead adipocytes were replaced with new ones. In the central necrotic zone, both adipocytes and adipose-derived stromal cells died. Thus, larger fat parcels will leave more tissue at risk of central necrosis and fibrosis. Fibrosis in thyroarytenoid muscle impedes mucosal wave movement, which is possibly the pathophysiology of the catastrophic “overinjection” complication [25] in AFIL.

Similar to previous work [29], we performed office-based structural AFIL in a multilayered and multiple injection point manner to avoid a large bolus injection at a single site. The average amount of fat tissue injected at each point was 0.13 mL in our study. Although their volume cannot be calculated, the fat parcels at each point will be even smaller. The contralateral healthy vocal fold was also injected with a smaller volume of fat tissue to enhance glottic closure.

The methodology of autologous fat harvesting has been long debated. A recent systematic review [35] failed to show a significant difference in outcomes of fat grafting among harvesting methods. Low negative pressure liposuction from the lower abdomen with centrifugation had been suggested to yield a larger stromal vascular fraction [36]. Our protocol was based on such evidence and is also more feasible for application in an office-based setting. Further studies are still needed in the future to understand the relationship between different techniques and grafted fat survival in AFIL.

While significant improvements were already noted at 2 weeks postoperatively, voice therapy administered afterwards was helpful for patients to adjust to the new vocal fold conditions and facilitate long-term recovery. Voice assessments, vocal hygiene, physical adjustments, and voice training are commonly addressed in voice therapy. Goals of voice therapy after surgery should be set according to the general wound healing stages: inflammation, proliferation and remodeling [37]. Vibration of the vocal folds was found to attenuate inflammation [38]. Gentle phonation, preferably in a semioccluded vocal tract, is utilized in the early stage of recovery [37,39]. Vocal exercises in the next stage promote oxygen transport and enhance hormonal release to mobilize fats and amino acids [37]. Voice activities of lower intensity are practiced and advised. In rehabilitative voice training, physician and speech language pathologists need to balance vocal performance with vocal fold impact to keep the recovering vocal fold from harm. For more efficient vocal output, a glottic posture of the vocal folds, barely touching a slightly open glottis, is more favorable [40]. In a later stage, when the vocal folds are robust, athletic vocal behaviors, such as extreme pitches and loudness, are trained. In voice rehabilitation, patients are trained to regain their vocal abilities, such as pitch range, loudness dynamic range, motor accuracy and agility, endurance, and other abilities specific to their individual needs.

Young et al. [41] pointed out that the minimal clinically important difference for improvement in the VHI-10 score in UVFP patients is a decrease of four. That is, a decrease of at least four is needed for UVFP patients to consider the improvement meaningful. In the current study, the VHI score improved by an average of 17.91 from preoperatively to 2 weeks postoperatively and another 6.36 from 2 weeks to 3 months postoperatively. All patients experienced an overall improvement significantly more than four. This indicates that the improvement is not only statistically significant but also clinically meaningful for every individual patient in our cohort. However, the VHI-10 score was not restored to the normal range in all patients. Radiotherapy and chemotherapy among lung cancer patients will cause dysphonia and hoarseness [42], even without UVFP. Nevertheless, we still believe office-based structural AFIL is a meaningful treatment for these patients since UVFP has been proven to be related to pneumonia in nationwide research [43]. Due to the high feasibility, office-based structural AFIL can easily be performed before or between cancer treatments without delay.

While the percentage differs between articles, thyroid surgery [2,3] is a well-known etiology of UVFP. Various techniques, such as intraoperative identification of the recurrent laryngeal nerve [44], have been developed to prevent UVFP in thyroidectomy. Despite the increasing use of recurrent laryngeal nerve monitoring to help identify the nerve, the incidence of unexpected complete vocal fold paralysis is still approximately 1.6% [45]. A large portion of our patients suffered from UVFP due to thyroid-related issues. Previous studies [46] have pointed out that injection laryngoplasty may improve voice and voice-related quality of life among post-thyroidectomy UVFP patients. Early injection laryngoplasty with long-lasting fillers [47] has recently been recommended by scholars for this group of patients. While proper human studies are difficult to design, a recent animal study [48] performed using recurrent laryngeal nerve resection swine also demonstrated that early injection with calcium hydroxyapatite may improve biomechanical properties and slow thyroarytenoid muscle atrophy compared to the uninjured vocal fold, which is comparable to our protocol and results.

Although autologous fat grafts have historically [49] been considered a temporary filler for injection laryngoplasty, more recent research has revealed promising long-term effects. In a meta-analysis performed in 2021 [22], Chang et al. pointed out that AFIL may improve the VHI score, GRBAS grade, and maximum phonation time for at least 12 months. By following the structural fat-grafting technique under general anesthesia, Cantarella et al. [29] presented a large series of AFIL patients with promising long-term effects for from 5 to 10 years. In patients who volunteered for further imaging, CT and serial MRI provided evidence for fat-graft survival with limited long-term fat absorption. While office-based structural AFIL might also have long-term effects among unilateral vocal paralysis patients, we designed a much shorter follow-up duration due to difficulties during the COVID-19 pandemic and lockdown. Further investigation will be needed in the future to demonstrate this assumption.

There are several other limitations of our study. First, acoustic characteristics, including fundamental frequency, jitter, and shimmer, were not recorded in our cohort due to lack of feasibility at our institution. These data may provide valuable information and be arranged in our future studies. Second, the injection of fat tissue was manually controlled in our research. The exact size and amount of each fat parcel cannot be recorded by this method. Modern fat-injection devices, such as the MAFT gun [50], can provide fat parcels smaller than 1/60 mL in a standard size, which may improve fat-grafting survival. Third, patients with a history of surgery for laryngeal and hypopharyngeal cancer surgery or a history of radiation were excluded from our study. The cover and body of the vocal folds [51] in these patients may have sustained various degrees of damage caused by radiotherapy or surgery. Thus, the surgical outcome of AFIL in these patients may be different from that in our series. However, head and neck patients often suffer from trismus, causing difficulties in docking for direct laryngoscopy under general anesthesia. These patients could potentially benefit from office-based procedures. We will perform further investigations in this population in the future.

## 5. Conclusions

Office-based structural AFIL can be considered an effective treatment option for UVFP patients. The described technique provides a reliable office-based laryngeal procedure for harvesting and injecting autologous fat in a structural manner. Significant improvements in the VHI-10 score and perceptual voice measurements were noted in our study.

## Figures and Tables

**Table 1:** Patient characteristics.

	N = 23
**Age (years)**	
Mean ± SD	58.65 ± 13.77
Range	30 to 82
**Sex**	
Male	9 (39.1%)
Female	14 (60.9%)
**BMI (kg/m^2^)**	
Mean ± SD	26.75 ± 4.33
Range	20.86 to 34.48
**Palsy side**	
Left	13 (56.5%)
Right	10 (43.5%)
**Etiology**	
Thyroid related	11 (47.8%)
Pulmonary and mediastinum related	6 (26.1%)
Cardiovascular surgery	1 (4.3%)
Cervical spine surgery	1 (4.3%)
Brain surgery	1 (4.3%)
Idiopathic	3 (13.0%)

Abbreviation: BMI, body mass index.

**Table 2:** Fat Injection points and volume.

	Ipsilateral Mean ± SD	Contralateral Mean ± SD
**Injection points**		
Medial injection	3.22 ± 0.80	2.00 ± 0.74
Lateral injection	2.13 ± 0.46	0
**Injected fat volume** (mL)	0.79 ± 0.17	0.19 ± 0.11

Abbreviation: SD, standard deviation.

**Table 3:** Results of GRBAS evaluation.

	Mean ± SD			*p* Value		
	PreOP	2 Weeks	3 Months	PreOP vs. 2 Wk	2 Wk vs. 3 Mo	PreOP vs. 3 Mo
**Grade**	2.17 ± 0.76	1.74 ± 0.69	1.05 ± 0.65	*p* = 0.002 *	*p* < 0.0001 *	*p* < 0.0001 *
**Roughness**	1.35 ± 0.93	1.26 ± 0.54	0.91 ± 0.53	*p* = 0.604	*p* = 0.017 *	*p* = 0.056
**Breathiness**	1.65 ± 1.02	0.78 ± 0.90	0.45 ± 0.67	*p* = 0.001 *	*p* = 0.016 *	*p* < 0.0001 *
**Asthenia**	0.70 ± 0.93	0.22 ± 0.52	0.09 ± 0.29	*p* = 0.013 *	*p* = 0.162	*p* = 0.004 *
**Strain**	0.13 ± 0.46	0.04 ± 0.21	0.09 ± 0.43	*p* = 0.426	*p* = 0.665	*p* = 0.747

Abbreviation: SD, standard deviation. PreOP, preoperative. PostOP, Postoperative. Wk, weeks. Mo, months. * Represents *p* < 0.05.

## Data Availability

Data generated are included within the manuscript.

## Supplementary Materials

The following supporting information can be downloaded at: https://www.mdpi.com/article/10.3390/jcm11164806/s1, Video S1: Office-based structural autologous fat injection laryngoplasty.
