# Supplementary material for: Office-Based Structural Autologous Fat Injection Laryngoplasty for Unilateral Vocal Fold Paralysis
Source: J Clin Med. 2022 Aug 17;11(16):4806. doi: 10.3390/jcm11164806 (PMC9410197; doi:10.3390/jcm11164806)
Supplement: Supplementary file 1 [file jcm-11-04806-s001.zip › supplementary.pdf]

Supplemental material

Video S1. Office-based structural autologous fat injection laryngoplasty

The current video clip demonstrates an example of office-based structural AFIL. Note the multiple injection point manner distributes purified fat tissue, tailored under real time endoscopic feedback.
